# Supplementary material for: Spatiotemporal spike-centered averaging reveals symmetry of temporal and spatial components of the spike-LFP relationship during human focal seizures
Source: Commun Biol. 2023 Mar 25;6:317. doi: 10.1038/s42003-023-04696-3 (PMC10039941; doi:10.1038/s42003-023-04696-3)
Supplement: Supplementary file 2 — Supplementary Information [file 42003_2023_4696_MOESM2_ESM.pdf]

Supplementary Information

Spatiotemporal spike-centered averaging reveals symmetry of temporal and spatial components of the spike-LFP relationship during human focal seizures

Somin Lee, Sarita S. Deshpande, Edward M. Merricks, Emily Schlafly, Robert Goodman, Guy M. McKhann, Emad N. Eskandar, Joseph R. Madsen, Sydney S. Cash, Michel J.A.M. van Putten, Catherine A. Schevon, Wim van Drongelen

Supplementary Notes

Supplementary Note 1. The sine cardinal (sinc) function

The observation that the spatial average over a small-time interval resembles a sinc function in Patients 1-3 is used for the interpretation of the relationship between spatial and temporal STAs. The central part of the reasoning is that, in general, the relationship between a rectangular function and the sinc function (Supplementary Fig. 1) can be written in the form:

$$sinc(y) \propto \int_{-\infty}^{\infty} rect(-X, X)\, e^{jxy} dx \tag{S1}$$

Here x, y are a pair of dimensions (e.g., time and space or time and frequency) and $rect(-X, X)$ is a rectangular function over $(-X, X)$; $j = \sqrt{-1}$.

Based on the definition of the Fourier transform and its inverse, the sinc function and rectangle function are Fourier transform pairs[1].

The property in Eq. S1 is used to model the spatiotemporal relationship of the electrical activity recorded by a macroelectrode (Fig. 1). Because of Eq. S1, the spatiotemporal activity function in Fig. 1, $f(r, \tau)$ can be approximated by $e^{jr\tau}$. The spatiotemporal symmetry as outlined in the first section of the results follows from this. In summary, in this situation, spatial and temporal aspects of the ongoing activity under the macroelectrode are coupled.

Supplementary Note 2. The spatiotemporal spike-triggered-average (st-SCA) as a unit impulse response

Computation of the spatiotemporal spike-centered average (st-SCA) using ictal recordings presents a challenge because the occurrence of action potentials across a seizing network is not experimentally controlled, unlike the scenario in which the location and timing of the neuronal activities are evoked by external stimuli. The approach as outlined in Eqs. S2-S5 addresses this problem and demonstrates that the st-SCA is spatiotemporal analog of well-known spike-triggered average (STA).

For convenience, we repeat here that (x, y, t) are the spatiotemporal components of the signals; (x_i, y_i, t_i) are the spatiotemporal coordinates of spike i and (ξ, ψ, τ) are the spatiotemporal components of the signal relative to the spike. Using a similar approach as in Eissa et al. (2018)[2], we now extend the model of the ictal network as a linear time invariant (LTI) system with the multi-unit action potential activity as input, the LFP as its output (note that this LTI system isn't necessarily causal), and the network's unit impulse response (UIR) (see Main Text Eq. 5) defined as the LFP associated with a single unit impulse (δ):

$$UIR = st\text{-}SCA = C(\xi, \psi, \tau) \tag{S2}$$

We now can recover the network output Z using the convolution of the UIR and the network's input, i.e. the spikes:

$$Z = \iiint C(\xi, \psi, \tau) \left\{ \frac{1}{N} \sum_{i=1}^{N} \delta(x - x_i - \xi, y - y_i - \psi, t - t_i - \tau) \right\} d\xi \, d\psi \, d\tau \tag{S3}$$

Note that we used the $\frac{1}{N}$ scaled version of the input here. Plugging in the expression for $C(\xi, \psi, \tau)$ (see Eq. 11) results in:

$$Z = \iiint \left\{ \frac{1}{N} \sum_{i=1}^{N} LFP(x_i + \xi, y_i + \psi, t_i + \tau) \right\} \ldots$$

$$\ldots \left\{ \frac{1}{N} \sum_{i=1}^{N} \delta(x - x_i - \xi, y - y_i - \psi, t - t_i - \tau) \right\} d\xi \, d\psi \, d\tau \tag{S4}$$

Exchange of the summation and integration operations and evaluation of the triple integral gives the model's estimate of the spatiotemporal LFP from the LTI system:

$$Z = \frac{1}{N^2} \sum_{i=1}^{N} \sum_{i=1}^{N} LFP(x, y, t) \; = \; LFP(x, y, t) \tag{S5}$$

As shown in Eissa et al. (2018)[2], the time domain component of this linear estimate produces a close approximation of the ongoing seizure activity with significant correlation ($p < 0.01$) between recorded and estimated activity.

Supplementary Note 3. Mechanisms involved in focal seizures

By combining current and previous findings on ictal dynamics, we can outline the following summary for an evolving neocortical focal seizure. At the micro and meso-scales, an ictal wave of action potential activity propagates at a velocity of ~1 mm/s by invoking excitation via the local connections over distances < 1 mm. This wave of hyperexcitation propagates locally when the inhibition in front of this wave fails to constrain the excitation[3-5]. In this context, it is interesting to note that this propagation process seems compatible with the evolution of the clinically observed Jacksonian march first described by Hughlings Jackson in 1870[6]. We now find evidence that, in addition to the slow propagation process, the ictal wave excites cortical areas farther than 1 mm away, probably via axon collaterals within the gray matter, which allows excitation to 'escape,' and enables recruitment of additional cortical territory (Fig. 6C). This activation of areas > 1 mm away might also explain modular propagation of ictal activity, a property previously observed in experimental seizures[7]. At the macroscale, white matter intracortical connections are invoked, spreading ictal activity across a cm-sized territory. The activity in this macroscale territory is still highly correlated with the action potential activity in the ictal wave located in the recruited territory rather than the local action potential activity located in the non-recruited areas (Supplementary Fig. 5)[4]. In addition, while local inhibition fails at the ictal wavefront, longer range inhibition remains intact and plays a critical role in sustaining the synchronous oscillatory component of the ongoing seizure at the macroscale[2,4].

Supplementary Figures and Tables

Supplementary Figure 1. Simulated 1-dimensional (D) and 2D sinc functions.

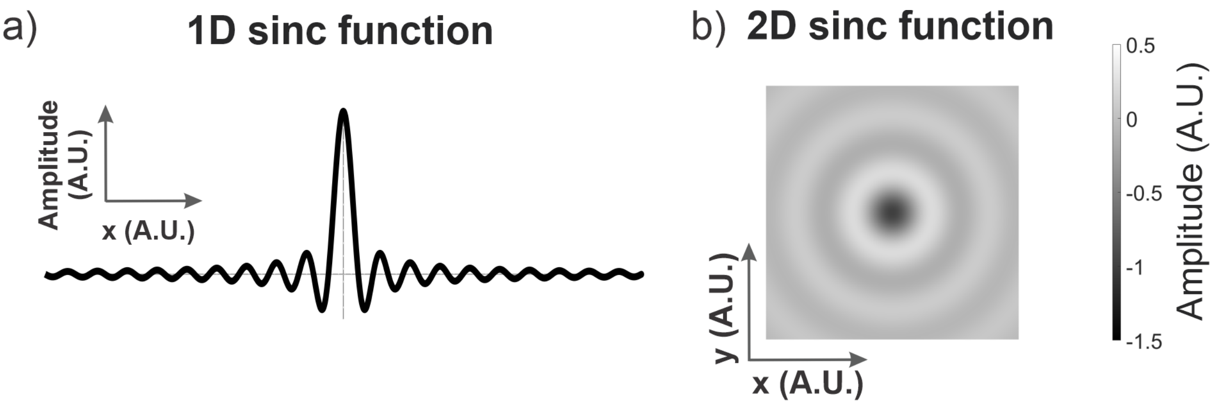

a) Simulation of a 1D sinc function, $sinc(x)$.
b) Top view of a simulated 2D sinc function.

Supplementary Figure 2: Representative temporal and spatial components of the spike-centered averages (SCAs) for each patient. Patients 1-5 had microelectrode arrays (MEAs) implanted in recruited territory, and Patients 6-7 had MEAs implanted in unrecruited territory. Grayscale is in μV units.

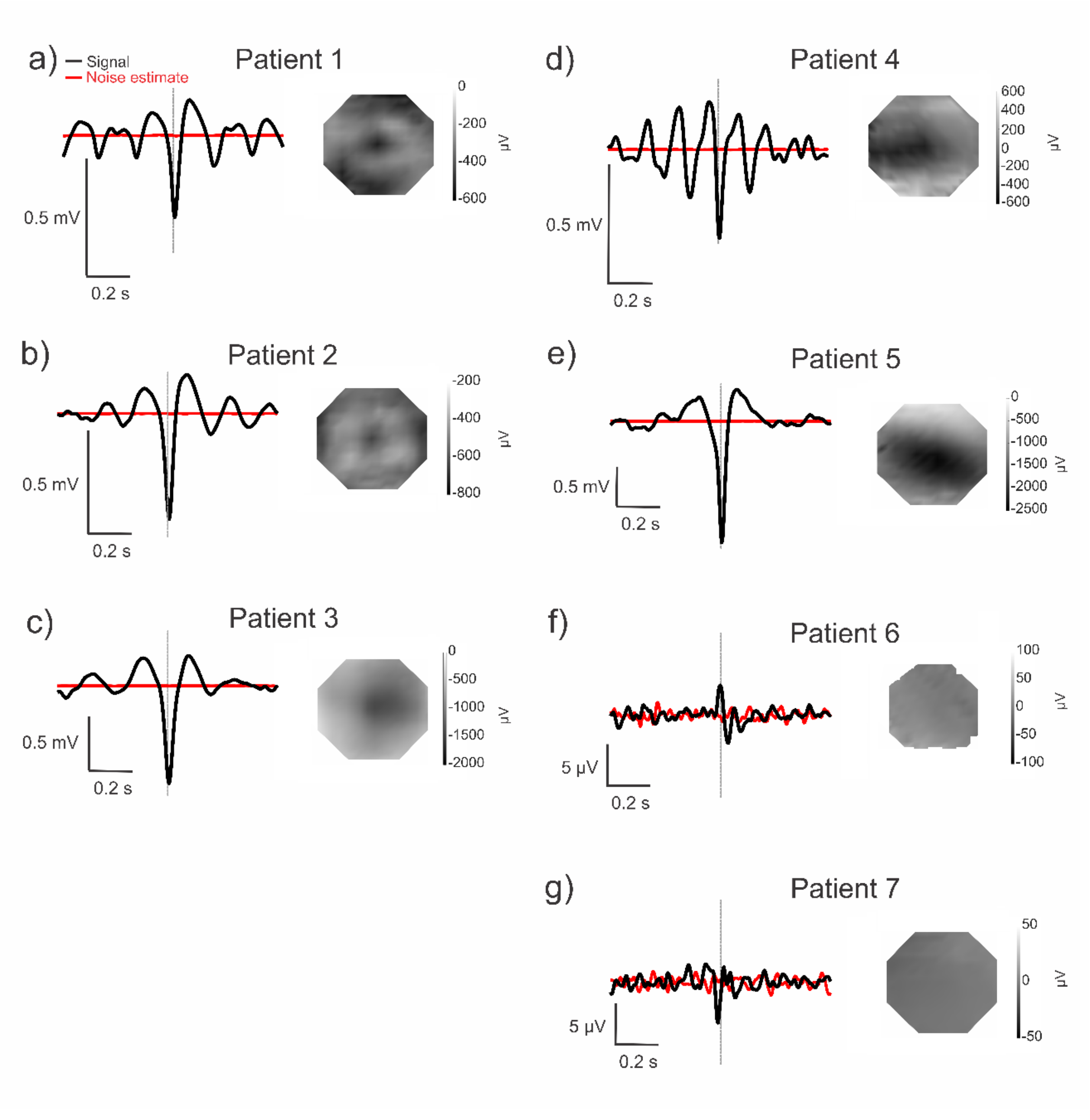

a—c) Patients 1-3 resemble sinc functions in the temporal and spatial domains. The black traces are the signals, and the red traces are the noise estimates.

d—e) Patients 4-5 do not resemble sinc functions in the temporal domain and resemble deep wells of in the spatial domain.

f—g) Patients 6-7 are characterized by comparatively much smaller amplitude signals in both the temporal and spatial components.

Supplementary Figure 3: Spatial component of the spatiotemporal spike-centered averages (st-SCAs) in recruited and unrecruited territories.

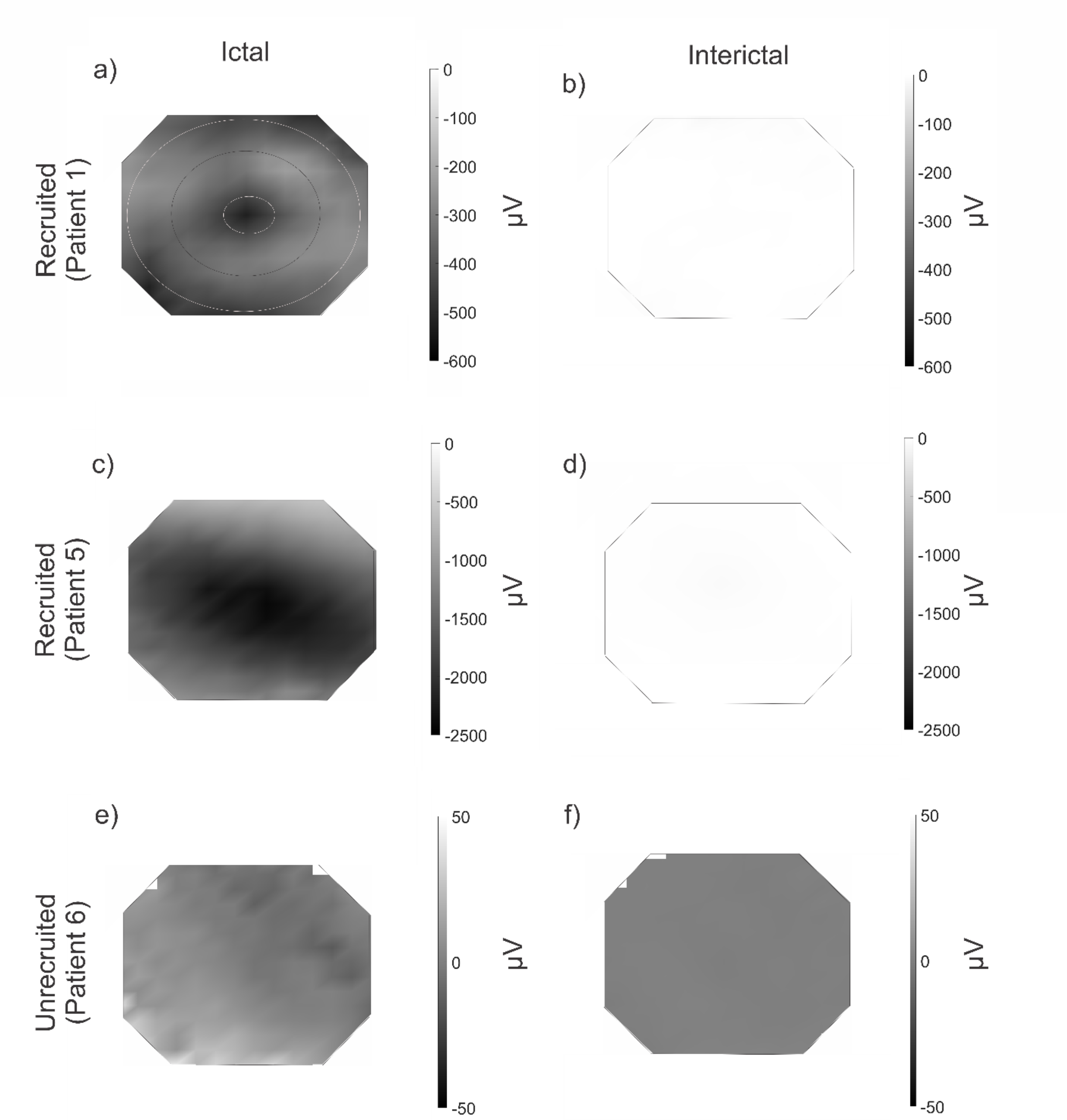

The st-SCA in panels a and c represent the same spike-LFP relationship as depicted in Fig. 4e and f, respectively. In all patients, the ictal signal (a, c, e) is stronger than the interictal one (b, d, f). In Patient 1 (a), the two rings surrounding the center are indicated by the circles. Patient 5 (c) instead shows a deep well of negative activity. The dynamics in unrecruited territories (e-f) are markedly different and are also much smaller in amplitude. Grayscale is in µV units.

Supplementary Figure 4: Noise estimates of the spatiotemporal spike-centered average (st-SCA) of Patient 1. The SNR of the signals is well above 14dB in each panel (as per application of the so-called five sigma rule)[8].

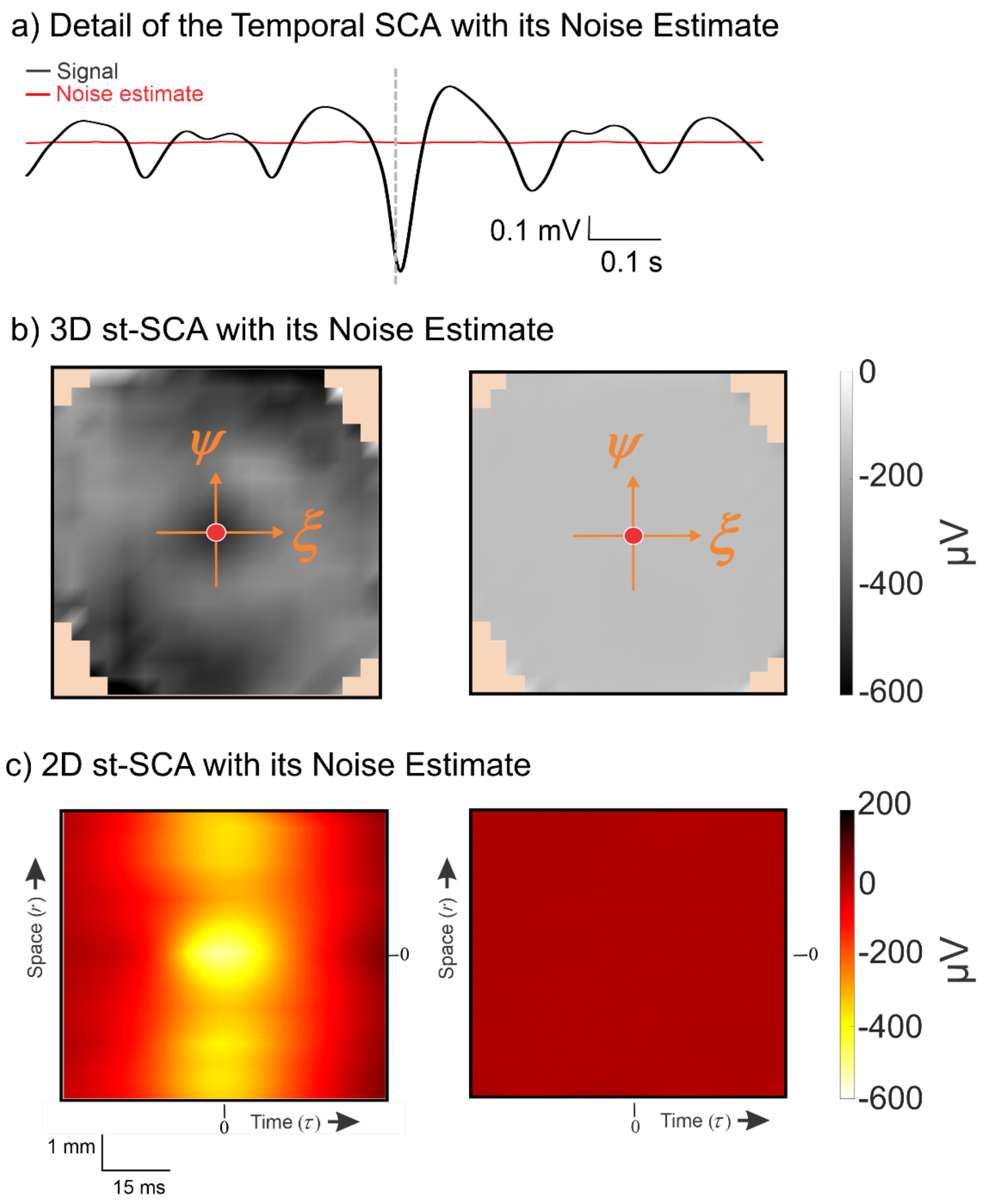

a) Detail of the temporal component of the st-SCA from Fig. 4c (black) and its noise estimate (red). The signal-to-noise ratio (SNR) of the depicted data is 45dB.

b) The spatial component of the st-SCA depicted in Fig. 4e and its estimated noise component. The location specific SNR of the depicted data range is 26 – 80dB, with an average of 38dB. The units for the

grayscale are identical for both maps and identical to the scale in Fig. 4e, Supplementary Fig. 2a, Supplementary Fig. 3a.

c) The 2D st-SCA from Fig. 5c and its noise estimate. The SNR of the depicted data is 39dB. The units for the color scale are identical for both maps and the same as in Fig. 5.

Supplementary Figure 5: Temporal spike-triggered averages (STAs) based on the recorded LFP in recruited and unrecruited territories averaged using spike triggers from locations in the recruited or unrecruited territory.

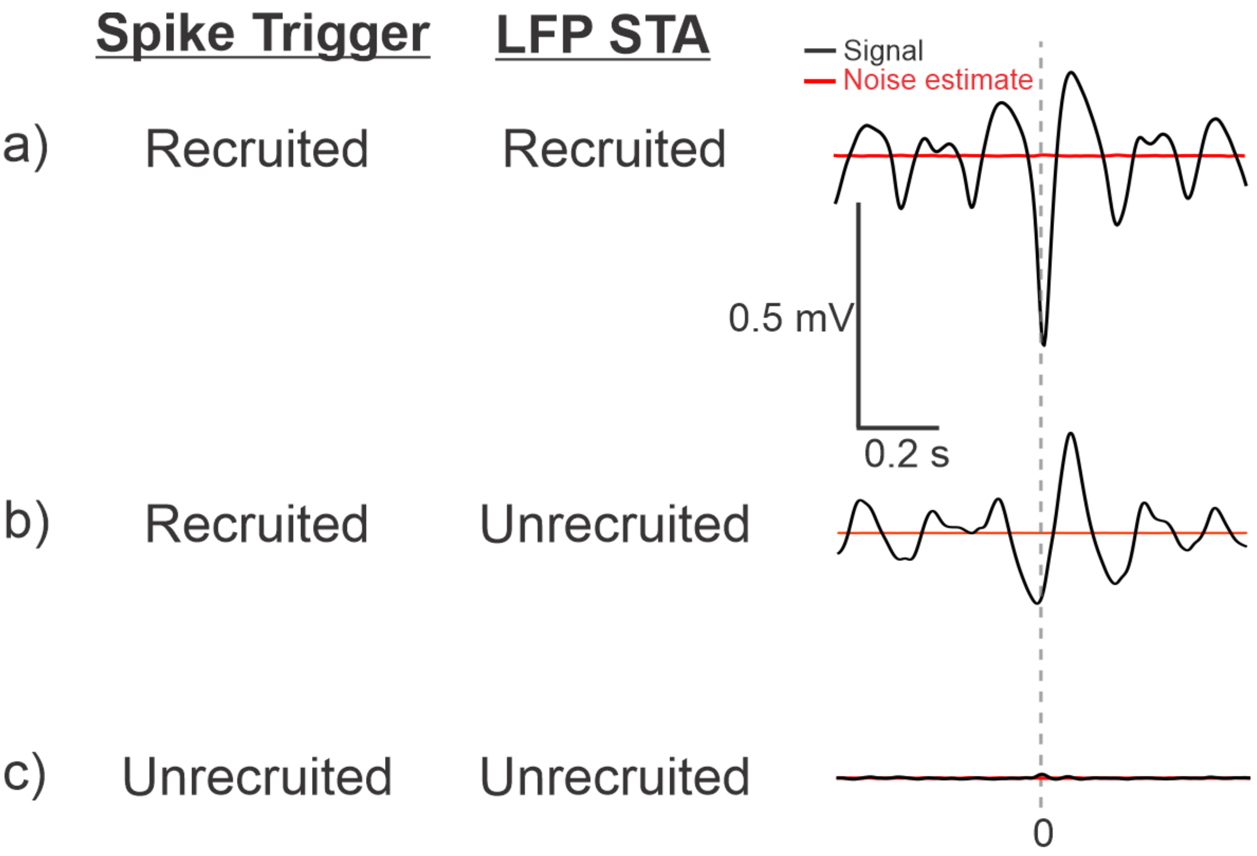

The STA of the LFP in the recruited area triggered by spikes in the recruited area (a) show a large negative peak at the time of the trigger. The STA in the unrecruited areas have a relatively strong signal component when triggered by spikes in from the recruited areas (b), but not if triggered by spikes in the unrecruited area (c). The black traces are the signals, and the red traces are the noise estimates.

Supplementary Figure 6. Representative spatiotemporal spike-centered averages (st-SCAs) after randomization of spike trigger timing.

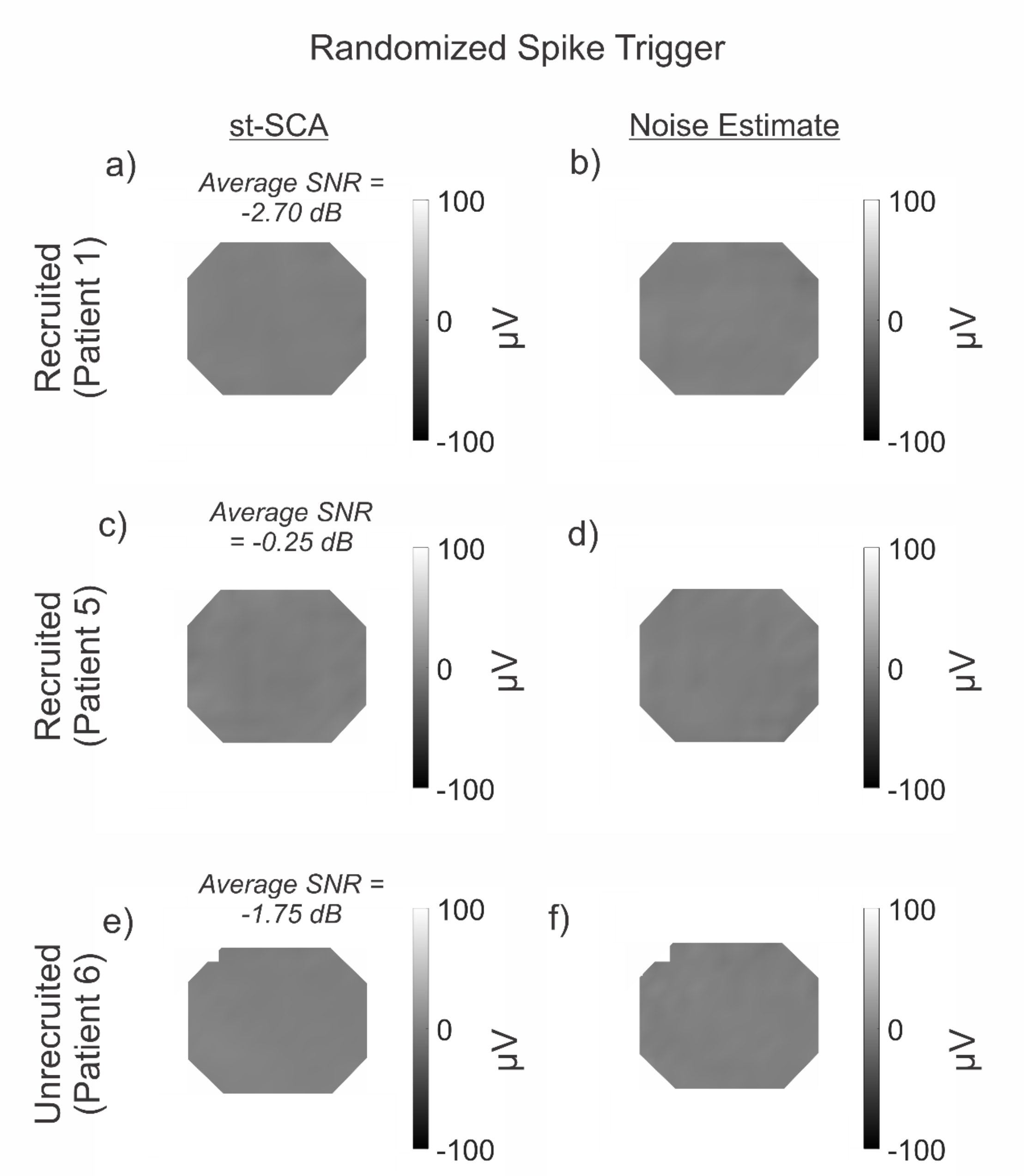

No spatial patterns are seen, highlighting the importance of spike timing in the st-SCA calculation. Grayscale is in arbitrary units (A.U.). The average signal-to-noise ratios (SNR) are listed per patient.

Supplementary Figure 7: List of representative spatiotemporal spike-centered averages (st-SCAs) after spatial filtering.

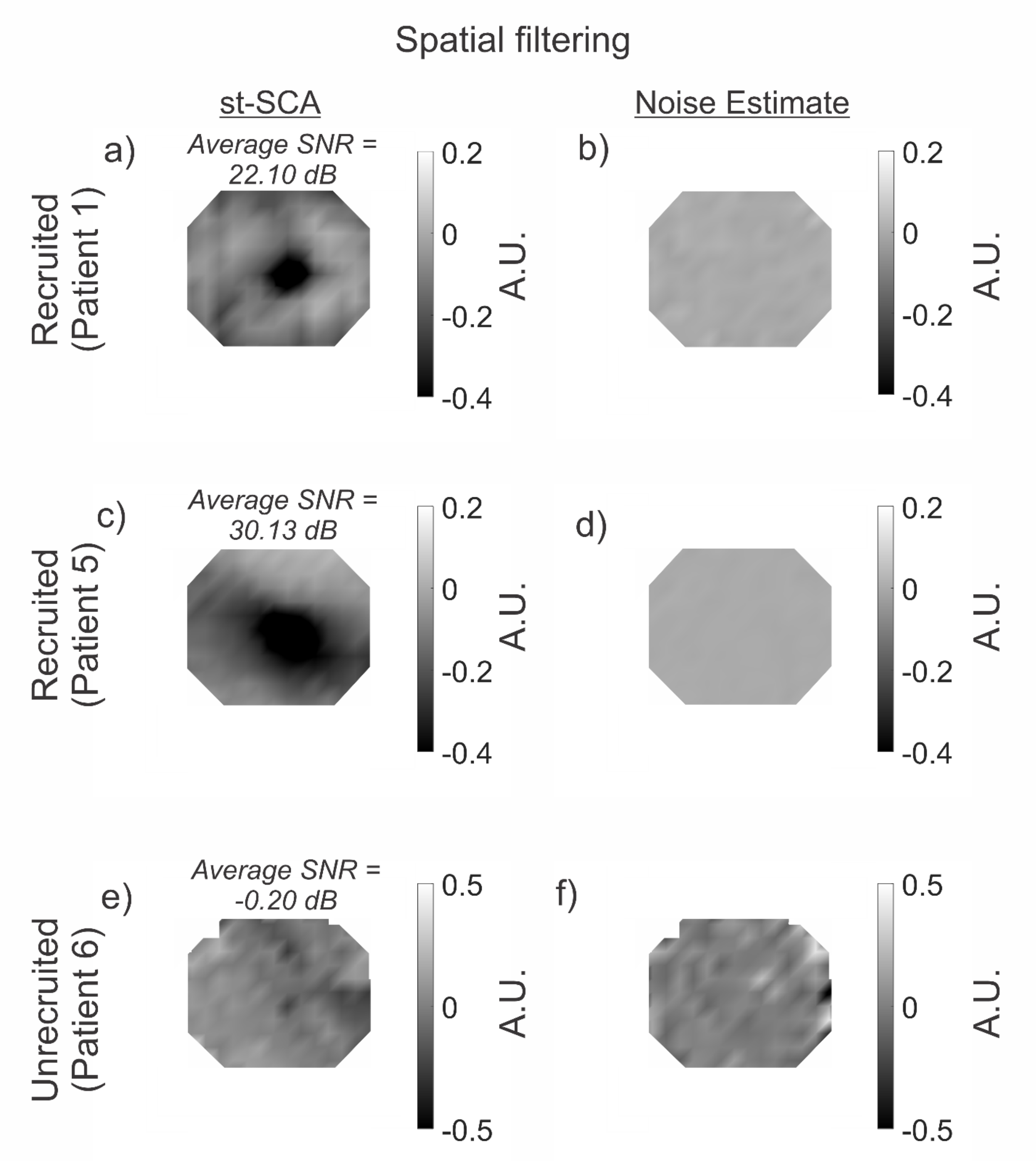

The spatially filtered st-SCAs resemble similar patterns to non-whitened st-SCAs, albeit a smaller amplitude signal. Grayscale is in arbitrary units (A.U.). The average signal-to-noise ratios (SNR) are listed per patient.

Supplementary Table 1. Patient Table: Demographics and Clinical Features

Patient (age/gender)	Implant location	MEA location	Seizure onset zone	No. of seizures analyzed	Seizure type(s)	Pathology
**Patient 1 (25yo/female)**	Left lateral and subtemporal	Left inferior temporal gyrus 2.5 cm from anterior temporal pole	Left basal/anterior temporal	3	Complex partial	Mild CA1 neuronal loss; lateral temporal nonspecific
**Patient 2 (19yo/female)**	Right lateral and subtemporal, parietal, occipital	Right posterior temporal, 1 cm inferior to angular gyrus	Right posterior lateral temporal	1	Complex partial with secondary generalization	Nonspecific
**Patient 3 (21yo/male)**	Left lateral frontal, subfrontal, temporal, subtemporal	Left middle temporal gyrus 1–2 cm posterior to the temporal tip	Left mesial temporal	3	Complex partial	Moderate CA3 & CA4 neuronal loss and gliosis
**Patient 4 (32yo/male)**	Left lateral temporal, subtemporal, parietal, frontal	Left superior temporal gyrus	Left anterior fronto-temporal	3	Complex partial	Cortical dysplasia
**Patient 5 (45yo/male)**	Right lateral temporal, parietal, frontal	Right superior temporal gyrus	Right anterior temporo-parieto-occipital	3	Complex partial with secondary generalization	Nonspecific
**Patient 6 (30yo/male)**	Left lateral frontal, mesial frontal, temporal	Left supplementary motor area, 3 cm superior to Broca's area	Left supplementary motor area	3	Complex partial/tonic	N/A (multiple subpial transections performed)
**Patient 7 (39yo/male)**	Left lateral and mesial frontal	Left lateral frontal 2 cm superior to Broca's area	Left frontal operculum (3 × 3-cm cortical area)	3	Complex partial	Nonspecific

Supplementary Table 2. Patient Table: Seizure Recording and Spike Detection Information

	Epoch Length (sec)	n spikes*	spikes/s*
**PATIENT 1**			
Interictal	180	7720	43
Seizure 1	58	78479	1353
Seizure 2	80	77788	972
Seizure 3	102	153063	1501
**PATIENT 2**			
Interictal	180	181116	1006
Seizure 1	29	110896	3824
**PATIENT 3**			
Interictal	180	16582	92
Seizure 1	52	162707	3129
Seizure 2	88	274656	3121
Seizure 3	57	193733	3399
**PATIENT 4**			
Interictal**	180	23881	133
Seizure 1	82	385978	4707
Seizure 2	102	366705	3595
Seizure 3	96.23	322148	3348
**PATIENT 5**			
Interictal	180	52998	294
Seizure 1	102	304058	2981
Seizure 2	101	314402	3113
Seizure 3	73	349189	4783
**PATIENT 6**			
Interictal	180	24438	136
Seizure 1	12	3471	289
Seizure 2	13	4902	377
Seizure 3	6	1635	273
**PATIENT 7**			
Seizure 1	20	17157	858
Seizure 2	23	7778	338
Seizure 3	31	7065	228

*Across all channels of the MEA.

**Due to limitations in available recordings, this interictal clip is 12 minutes away from the nearest known ictal activity

Supplementary Table 3. Spatiotemporal spike-centered average (st-SCA) contributions per each position of the 19x19 grid for Patient 1.

0	0	0	103	255	820	2455	3733	3820	3843	3843	3740	3588	3023	1388	110	23	0	0
0	0	103	1351	1916	4367	6910	8496	8519	9601	9646	8550	8447	7479	5649	2515	1237	68	0
0	0	1295	2528	5459	8359	12487	14617	17793	19560	21165	20084	17335	17183	14451	9508	4660	2851	45
0	967	3247	5426	6807	11063	16262	18528	21611	24829	25761	25188	22908	21201	19441	15082	10006	5505	1746
871	967	3724	6357	7885	12650	17621	20154	25398	29523	28658	27631	24732	22895	21466	16717	11715	7854	2678
871	3287	9713	13611	15223	20139	25265	27873	34893	37525	34148	31846	27605	24035	21722	18477	14282	9613	2684
3191	8533	14665	18792	21507	26302	32201	35560	45834	48131	45108	38963	32494	29372	26614	23873	17695	12947	2974
5699	11151	19184	22539	25680	32674	39669	45411	56147	60608	52622	48376	40281	36785	29212	24439	18118	14293	3488
7927	12450	21762	26520	30323	38476	46592	52892	65817	71443	62671	54461	49289	39763	34507	26143	19817	12622	6087
9127	13359	22366	27238	30418	39017	48327	56367	65787	78479	63226	57602	48841	43962	33058	30803	19025	13447	5318
9127	13528	21382	27053	29659	35132	46226	53925	60109	64798	55277	51414	46905	39197	32124	27793	17662	9285	4057
9127	13432	19430	24503	29167	34290	37810	45054	51606	60047	50782	46523	44215	37950	29328	24965	13723	5175	2216
8256	13432	19728	23326	25981	28722	31439	40238	40611	48968	39481	36527	32763	25651	22666	18687	11858	2283	2176
8256	12561	15606	19098	23375	27189	29788	35703	36666	40196	30809	29173	24897	21777	13927	12645	8363	3189	1875
5936	9823	13364	20424	21921	25322	29748	36538	31736	32811	27806	24121	22046	18885	14415	11512	7097	1876	6
3428	7113	6394	12652	16398	17789	20958	27735	25045	25668	24040	18424	19431	16942	14840	6899	4921	112	0
1200	4506	4055	5418	8778	12290	14600	19003	15726	16523	17926	16363	11102	9996	6798	3764	1987	0	0
0	1369	2390	2773	3716	5793	7012	10406	11718	9940	9776	8955	7851	4999	2674	113	6	0	0
0	0	169	281	295	592	1213	1283	2152	2089	1977	1963	1666	1045	975	106	0	0	0

Supplementary Table 4. Signal-to-noise ratio (SNR; dB) values per each position of the 19x19 grid for Patient 1. The pixels with SNR values that do not satisfy the Rose criterion are indicated in bold.

**NaN**	**NaN**	**NaN**	18	29	29	34	40	38	39	38	34	32	29	26	19	**3**	**NaN**	**NaN**
**NaN**	**NaN**	17	34	42	44	46	57	54	50	49	50	47	54	56	35	35	19	**NaN**
**NaN**	**NaN**	44	63	41	45	61	62	53	49	41	40	39	41	40	45	36	36	**4**
**NaN**	48	74	45	44	58	60	58	39	37	40	40	37	41	46	41	38	36	27
37	40	54	37	45	52	44	55	40	38	38	38	36	38	45	43	35	33	34
35	34	37	38	40	45	46	44	34	35	36	36	36	39	43	40	39	34	36
33	40	41	44	48	48	50	42	37	36	36	36	39	40	65	48	37	40	42
36	38	45	38	44	43	52	46	40	41	41	44	50	42	49	42	40	36	38
53	40	43	43	45	51	62	52	43	46	47	48	46	53	58	46	42	39	41
45	39	48	44	45	51	56	56	46	48	54	55	51	52	41	50	40	46	39
44	43	48	46	47	56	57	53	49	48	44	44	61	42	51	56	43	40	36
47	45	38	42	52	58	42	47	67	48	54	48	46	48	49	44	40	40	33
82	43	39	50	51	44	43	55	43	43	43	39	40	42	42	88	36	36	55
54	38	39	41	44	67	43	57	58	51	51	43	60	51	47	43	45	46	34
38	41	35	44	43	43	65	55	46	42	46	50	62	59	60	50	57	36	**4**
33	48	41	51	51	55	43	50	68	52	68	44	43	59	46	39	43	35	**NaN**
27	44	40	37	43	50	49	47	55	63	49	44	49	64	46	48	37	**NaN**	**NaN**
**NaN**	30	37	35	51	36	34	40	44	42	44	53	47	46	46	37	**2**	**NaN**	**NaN**
**NaN**	**NaN**	25	33	46	39	32	27	29	27	30	31	29	27	29	51	**NaN**	**NaN**	**NaN**

Supplementary References

1 Boashash, B. in *Time-Frequency Signal Analysis and Processing (Second Edition)* (ed Boualem Boashash) 3-29 (Academic Press, 2016).

2 Eissa, T. L. *et al.* The Relationship Between Ictal Multi-Unit Activity and the Electrocorticogram. *Int J Neural Syst* **28**, 1850027, doi:10.1142/S0129065718500272 (2018).

3 Schevon, C. A. *et al.* Evidence of an inhibitory restraint of seizure activity in humans. *Nat Commun* **3**, 1060, doi:10.1038/ncomms2056 (2012).

4 Eissa, T. L. *et al.* Cross-scale effects of neural interactions during human neocortical seizure activity. *Proc Natl Acad Sci U S A* **114**, 10761-10766, doi:10.1073/pnas.1702490114 (2017).

5 Tryba, A. K. *et al.* Role of paroxysmal depolarization in focal seizure activity. *J Neurophysiol* **122**, 1861-1873, doi:10.1152/jn.00392.2019 (2019).

6 Extercatte, J., de Haan, G. J. & Gaitatzis, A. Teaching Video NeuroImages: Frontal opercular seizures with jacksonian march. *Neurology* **84**, e83-84, doi:10.1212/wnl.0000000000001363 (2015).

7 Trevelyan, A. J., Sussillo, D., Watson, B. O. & Yuste, R. Modular propagation of epileptiform activity: evidence for an inhibitory veto in neocortex. *J Neurosci* **26**, 12447-12455, doi:10.1523/JNEUROSCI.2787-06.2006 (2006).

8 Rose, A. & Rose, A. Human vision. *Vision: Human and Electronic*, 29-53 (1973).
