# Supplementary material for: Spatiotemporal spike-centered averaging reveals symmetry of temporal and spatial components of the spike-LFP relationship during human focal seizures
Source: Commun Biol. 2023 Mar 25;6:317. doi: 10.1038/s42003-023-04696-3 (PMC10039941; doi:10.1038/s42003-023-04696-3)
Supplement: Supplementary file 3 — Description of Additional Supplementary Files [file 42003_2023_4696_MOESM3_ESM.pdf]

Description of Additional Supplementary Files

File name: Supplementary Movie 1

Description: This video shows the multi-unit activity depicting the propagation of the ictal wavefront and the associated 2-50 Hz low frequency component of the LFP from the MEA from Patient 1. The top trace shows summed LFP activity from all MEA channels with the vertical red line noting the current time in the recording. Seizure onset is at 15 seconds, and seizure termination is at 73 seconds.
