# Supplementary material for: Spatiotemporal spike-centered averaging reveals symmetry of temporal and spatial components of the spike-LFP relationship during human focal seizures
Source: Commun Biol. 2023 Mar 25;6:317. doi: 10.1038/s42003-023-04696-3 (PMC10039941; doi:10.1038/s42003-023-04696-3)
Supplement: Supplementary file 5 — Reporting summary [file 42003_2023_4696_MOESM5_ESM.pdf]

Corresponding author(s): Wim van Drongelen

Last updated by author(s): 03/03/2023

Reporting Summary

Nature Portfolio wishes to improve the reproducibility of the work that we publish. This form provides structure for consistency and transparency in reporting. For further information on Nature Portfolio policies, see our Editorial Policies and the Editorial Policy Checklist.

Statistics

For all statistical analyses, confirm that the following items are present in the figure legend, table legend, main text, or Methods section.

n/a	Confirmed	
☐	☒	The exact sample size (n) for each experimental group/condition, given as a discrete number and unit of measurement
☐	☒	A statement on whether measurements were taken from distinct samples or whether the same sample was measured repeatedly
☒	☐	The statistical test(s) used AND whether they are one- or two-sided<br>*Only common tests should be described solely by name; describe more complex techniques in the Methods section.*
☒	☐	A description of all covariates tested
☐	☒	A description of any assumptions or corrections, such as tests of normality and adjustment for multiple comparisons
☒	☐	A full description of the statistical parameters including central tendency (e.g. means) or other basic estimates (e.g. regression coefficient) AND variation (e.g. standard deviation) or associated estimates of uncertainty (e.g. confidence intervals)
☒	☐	For null hypothesis testing, the test statistic (e.g. F, t, r) with confidence intervals, effect sizes, degrees of freedom and P value noted<br>*Give P values as exact values whenever suitable.*
☒	☐	For Bayesian analysis, information on the choice of priors and Markov chain Monte Carlo settings
☒	☐	For hierarchical and complex designs, identification of the appropriate level for tests and full reporting of outcomes
☒	☐	Estimates of effect sizes (e.g. Cohen's d, Pearson's r), indicating how they were calculated

Our web collection on statistics for biologists contains articles on many of the points above.

Software and code

Policy information about availability of computer code

Data collection	All EEG recordings were obtained through 4mm X 4mm Utah microelectrode arrays by Blackrock systems alongside clinical probes.
Data analysis	MATLAB was used for all analyses presented in this study. Custom code will be made available in a public repository.

For manuscripts utilizing custom algorithms or software that are central to the research but not yet described in published literature, software must be made available to editors and reviewers. We strongly encourage code deposition in a community repository (e.g. GitHub). See the Nature Portfolio guidelines for submitting code & software for further information.

Data

Policy information about availability of data

All manuscripts must include a data availability statement. This statement should provide the following information, where applicable:

- Accession codes, unique identifiers, or web links for publicly available datasets
- A description of any restrictions on data availability
- For clinical datasets or third party data, please ensure that the statement adheres to our policy

The data used for this study are available on request from the corresponding author (W.v.D). The data is not publicly available due to institutional IRB protocols, and all data sharing will have to follow protocols compliant with approved IRB

Field-specific reporting

Please select the one below that is the best fit for your research. If you are not sure, read the appropriate sections before making your selection.

☒ Life sciences ☐ Behavioural & social sciences ☐ Ecological, evolutionary & environmental sciences

For a reference copy of the document with all sections, see nature.com/documents/nr-reporting-summary-flat.pdf

Life sciences study design

All studies must disclose on these points even when the disclosure is negative.

Sample size	Sample size was determined by the number of recordings available from epilepsy patients implanted with microelectrode arrays.
Data exclusions	EEG recordings were excluded only due to excessive artifacts and noise.
Replication	Results were replicated across two data collection sites (Columbia University and Harvard University).
Randomization	Randomization is not possible since surgical implantation of microelectrode arrays can only be performed on pre-screened epilepsy patients meeting a series of criteria.
Blinding	Blinding not possible or necessary since the data collection involved surgical implantation of microelectrode arrays in patients identified to have neocortical temporal lobe epilepsy.

Reporting for specific materials, systems and methods

We require information from authors about some types of materials, experimental systems and methods used in many studies. Here, indicate whether each material, system or method listed is relevant to your study. If you are not sure if a list item applies to your research, read the appropriate section before selecting a response.

Materials & experimental systems

n/a	Involved in the study	
☒	☐	Antibodies
☒	☐	Eukaryotic cell lines
☒	☐	Palaeontology and archaeology
☒	☐	Animals and other organisms
☐	☒	Human research participants
☒	☐	Clinical data
☒	☐	Dual use research of concern

Methods

n/a	Involved in the study	
☒	☐	ChIP-seq
☒	☐	Flow cytometry
☒	☐	MRI-based neuroimaging

Human research participants

Policy information about studies involving human research participants

Population characteristics	Patients with medically intractable neocortical focal epilepsy undergoing intracranial EEG monitoring for identification of the epileptogenic zone.
Recruitment	Cases that were considered appropriate included temporal lobe epilepsy, in which the implantation was performed to define the contribution of lateral temporal neocortex and tailor/extend temporal lobectomy, and extratemporal syndromes in which scalp EEG recording indicated a consistent and well-defined interictal and ictal focus limited to a sublobar distribution and confirmed by a neuroimaging study.
Ethics oversight	Procedures were approved by the Internal Review Board committees at Columbia University Medical Center, The University of Chicago Comer Children's Hospital, and Massachusetts General Hospital/Brigham and Women's Hospitals. The patients' surgeries and treatment plans were not directed by or altered as a result of these studies.

Note that full information on the approval of the study protocol must also be provided in the manuscript.
